# Seroprevalence of celiac disease antibodies in blood donors

**DOI:** 10.5339/qmj.2025.80

**Published:** 2025-09-10

**Authors:** Muhammad Umar Saddique, Abdelatif Al. Ahmed Abdelmola, Saloua Mohammed M. Hmissi, Alshimaa Alaboudy, Prem Chandra, Moutaz Farouk Mahmoud Derbala

**Affiliations:** 1 Department of Gastroenterology, Hamad Medical Corporation, Doha, Qatar; 2 Blood Donation Center, Hamad Medical Corporation, Doha, Qatar; 3 Department of Tropical Medicine and Gastroenterology, Sohag University, Siham Egypt; 4 Medical Research Center Hamad Medical Corporation, Doha, Qatar *Email: umar20008@gmail.com

**Keywords:** Celiac disease, anti-endomysial antibody, anti-tissue transglutaminase antibody, blood donors

## Abstract

**Background:**

Celiac disease (CD) is a chronic immune-mediated disorder of the digestive system triggered by gluten ingestion among genetically predisposed individuals. It is a chronic disease that causes malnutrition, but it is a non-fatal condition. A significant proportion of cases remain undiagnosed due to asymptomatic or nonspecific presentations, contributing to delayed diagnosis and long-term complications. CD is not a contraindication for blood donation. This study aims to determine the seroprevalence of CD antibodies and the prevalence of biopsy-proven CD among blood donors.

**Methods:**

A cross-sectional study was conducted on 1,497 blood donors (≥18 years) at Hamad Medical Corporation, Doha, Qatar, between 2018 and 2022. Serum anti-tissue transglutaminase IgA (anti-tTG IgA) and anti-endomysial IgA (anti-EM IgA) antibodies were measured in enrolled blood donors. Individuals with positive results for either antibody underwent esophagogastroduodenoscopy (EGD) with duodenal biopsies to confirm the diagnosis.

**Results:**

Serological testing revealed a seroprevalence of 0.8% (95% CI, 0.4%–1.4%) for anti-tTG IgA, with 83.3% of these individuals also being positive for anti-EM IgA. All seropositive individuals were male. Of 12 seropositive individuals, 4 (33%) consented to EGD and duodenal biopsy. Histological findings confirmed CD in two cases (50% of seropositive individuals who consented to biopsy). Both confirmed cases exhibited Marsh grade 2 changes.

**Conclusion:**

The seroprevalence of CD antibodies among healthy blood donors was 0.8%, and biopsy-proven CD was 50% among seropositive individuals who consented to EGD. These findings highlight the presence of undiagnosed CD in this population, predominantly in males, and underscore the need for further community-based studies to assess the true prevalence and clinical implications of CD.

## 1. BACKGROUND

Celiac disease (CD) is a chronic immune-mediated enteropathy triggered by gluten ingestion in genetically predisposed individuals. Its clinical manifestations are highly variable, ranging from classic gastrointestinal symptoms such as abdominal pain, diarrhea, and weight loss to extraintestinal complications like anemia, fatigue, and osteoporosis.^1^ A substantial proportion of individuals with CD, however, remain asymptomatic or present with nonspecific symptoms, leading to delayed diagnosis.^2^ This asymptomatic presentation represents a critical challenge for early detection and prevention of long-term complications associated with CD. The global prevalence of CD varies widely. In Europe and North America, the prevalence is estimated to be around 1%, whereas in the Middle East and South Asia, studies report rates between 0.5% and 0.9%. Despite the recognized burden of CD in neighboring regions, there is a paucity of data on its prevalence in the Gulf region, including Qatar.^3^ CD diagnosis relies on a two-step process: serum serological screening using anti-tissue transglutaminase IgA (anti-tTG IgA) and anti-endomysial IgA (anti-EM IgA antibodies), followed by duodenal biopsy for histological confirmation. Anti-tTG IgA has 90% to 98% sensitivity and 95% to 97% specificity. Anti-EM IgA has 85% to 98% sensitivity and 97% to 100% specificity.^4,5^ While serological tests provide a non-invasive method for initial screening, histological examination of duodenal biopsies remains the diagnostic gold standard, allowing for classification based on the Marsh criteria, which classify the severity of CD based on the degree of villous atrophy and intraepithelial lymphocytes.^6,7^ Serology is highly sensitive and specific. However, false-positive or false-negative results may occur. This is a major limitation when diagnosing CD based solely on serology. Some individuals with serological positivity do not show histological changes, possibly due to patchy mucosal involvement. Conversely, a subset of seronegative individuals may exhibit histological features of CD. In those cases, selective IgA deficiency should be ruled out, and serum anti-tTG IgG can help in the diagnosis. Some conditions cause villous atrophy in the absence of CD, such as severe combined immunodeficiency, Whipple’s disease, giardiasis, and non-steroidal anti-inflammatory drugs. Serology and HLA-DQ2/DQ8 testing can help differentiate those conditions from CD. Screening healthy populations, such as blood donors, can identify asymptomatic individuals who may otherwise remain undiagnosed.^8,9^ CD is not a contraindication for blood donation; there is no evidence of blood transfusion–related transmission.^10^ Previous studies conducted in various countries have leveraged blood donor screening to investigate CD prevalence, highlighting significant geographic and population-specific variations.^11–14^ This study aimed to assess the seroprevalence of CD antibodies and the prevalence of biopsy-confirmed CD among adult blood donors. By combining serological screening with biopsy, this research provides critical insights into the burden of undiagnosed CD in this population and addresses the gap in epidemiological data.

## 2. METHODS

This cross-sectional study was conducted at the Blood Bank of Hamad Medical Corporation (HMC), Doha, Qatar, the main secondary and tertiary health care provider, in collaboration with the Department of Gastroenterology, between 2018 and 2022. Blood donors aged ≥18 years who consented to participate were included. Pregnancy was an exclusion criterion. This study received approval from the Institutional Review Board through the Medical Research Centre (MRC-01-18-081). Participants were informed about the study objectives and procedures, and informed written consent was obtained. Data were collected through electronic chart review. Serological testing for anti-tTG IgA was performed using an enzyme-linked immunosorbent assay (ELISA), whereas anti-EM IgA was detected using indirect immunofluorescence. The ELISA kits used were obtained from Biomatik Medical™, Ontario, Canada. Antibody levels were quantified using a calibration curve and expressed in enzyme units per milliliter (U/mL), with interpretation criteria as follows: negative (<7 U/mL), equivocal (7–10 U/mL), and positive (>10 U/mL). Seropositive participants were contacted via phone, referred for a clinical evaluation, and were offered esophagogastroduodenoscopy (EGD) with duodenal biopsy. Biopsy samples were obtained from the duodenal bulb and the second portion of the duodenum and analyzed by an expert pathologist. Findings were classified according to the Marsh criteria.^6^ Complete blood count and ferritin levels were also assessed. Statistical analysis was performed using IBM SPSS version 29.0. Descriptive statistics were used to calculate the prevalence of CD antibodies and biopsy-confirmed CD; results are expressed as percentages with 95% confidence intervals (CIs).

## 3. RESULTS

A total of 1,497 healthy blood donors were screened, 1,296 males (86.6%) and 201 females (13.4%). Anti-tTG IgA antibodies were detected in 12 cases (0.8% [95% CI, 0.4%–1.4%), and anti-EM IgA antibodies were detected in 10 cases (0.67% [95% CI, 0.36%–1.23%). Two cases tested positive for anti-tTG IgA and negative anti-EM IgA antibodies. All 12 individuals (100%) were males. Four individuals (33.3%) who were positive for both antibodies consented to EGD and biopsy. Histological analysis confirmed Marsh grade 2 CD in two cases (50%) and peptic duodenitis in the remaining two cases. None of the participants reported symptoms, and no significant association was observed between seropositivity and blood type. The mean age of these individuals was 38 years (range: 35–42 years). The mean BMI was 28 kg/m² (range: 26–30 kg/m²), the mean hemoglobin level was 13.5 g/dL (range: 13.0–14.0 g/dL), and the mean ferritin level was 125 ng/mL (range: 30–400 ng/mL; Table 1).

## 4. DISCUSSION

Screening healthy blood donors for CD provides an estimate of its prevalence in the community. Large-scale community screening is not possible due to the silent nature of the disease and its nonspecific symptoms. These types of studies have been conducted across the world. Our study results align with global trends but highlight notable regional and demographic variations. The seroprevalence of CD antibodies in this study (0.8%) is slightly lower than in European populations, where rates range from 1% to 3%.^5,11^ However, it is comparable to findings in the Middle East, North Africa (0.6%), and South Asia (0.56%), suggesting shared genetic and environmental factors.^3,9,15^ A similar study in the United States examined 2,000 healthy blood donors. It found a 0.40% seropositivity rate, which is slightly lower than our study.^16^ All seropositive individuals in this study were male, which contrasts with global data that indicates a higher prevalence of CD in females.^17^ This discrepancy may be attributed to the male-dominated donor pool in blood donors (86.6%) and gender discrepancy in local demographics, where the male-to-female ratio is 3:1.^18^ This also highlights a similar trend of more frequent blood donation among males compared to females, which is a well-established fact.^19^ The low biopsy-confirmed prevalence in this study is likely influenced by the limited number of seropositive participants who consented to EGD (33%), a challenge observed in other similar studies.^12,20^ The absence of clinical symptoms in seropositive individuals reinforces the silent nature of CD, as most undiagnosed cases are asymptomatic. Elevated anti-tTG IgA titers in biopsy-confirmed cases support the high sensitivity and specificity of this serological marker.^20^ The relatively low number of seropositive individuals who underwent EGD (33%) likely contributed to the lower observed prevalence of biopsy-confirmed CD. These findings highlight the challenges of conducting invasive diagnostic procedures in asymptomatic individuals. Consequently, the true prevalence of CD in this population may be higher than indicated by biopsy-confirmed cases.^16,21^ Qatar’s diverse expatriate population, primarily from South Asia and the Middle East, highlights the need to understand local epidemiological patterns of CD. This study provides preliminary data indicating a high likelihood of undiagnosed CD in the community.^18^ This study has several limitations. The small number of seropositive individuals who consented to EGD limits the power to estimate the true prevalence of biopsy-confirmed CD accurately. The predominance of males in the study population restricts the generalizability of the findings. Additionally, the use of blood donors as the study population may introduce selection bias, as donors are typically healthier than the general population. These factors should be considered when interpreting the results, and future research should aim to include a more representative sample of the population.

## 5. CONCLUSION

This study found a 0.8% prevalence of CD antibodies among healthy blood donors. While this suggests undiagnosed CD in the community, it does not determine the overall community prevalence. The observed male predominance warrants further investigation to determine whether it reflects true trends or the composition of the donor pool. Larger, community-based studies are essential to estimate the true prevalence of CD. These efforts will guide public health strategies for early detection, management, and prevention of CD complications.

## LIST OF ABBREVIATIONS

**Table:** 

anti-EM IgA	Anti-endomysial IgA
anti-tTG IgA	Anti-tissue transglutaminase IgA
CD	Celiac disease
EGD	Esophagogastroduodenoscopy
ELISA	Enzyme-linked immunosorbent assay

## Figures and Tables

**Table 1.** Comparison of demographic and clinical characteristics among the total study population, seropositive individuals, and EGD participants.

**Characteristics**	**Total population (*N* = 1,497)**	**Seropositive individuals (*n* = 12)**	**EGD participants** **(*n* = 4)**
Gender			
Male	1,296 (86.6%)	12 (100%)	4 (100%)
Female	201 (13.4%)	0 (0%)	0 (0%)
Age (years)			
Mean (SD)	34.2 (8.5)	38 (3.2)	38 (2.8)
BMI (kg/m²)			
Mean (SD)	27.5 (4.2)	28 (1.8)	28 (1.8)
Height (cm)			
Mean (SD)	169.5 (6.3)	167 (2.4)	167 (2.4)
Hemoglobin (g/dL)			
Mean (SD)	13.5 (0.5)	13.5 (0.5)	13.5 (0.5)
Ferritin (ng/mL)			
Mean	125 (20)	125 (20)	125 (20)
Ethnicity			
South Asian	932 (62.3%)	7 (58.3%)	2 (50%)
Middle Eastern	402 (26.9%)	4 (33.3%)	1 (25%)
Other	163 (10.8%)	1 (8.4%)	1 (25%)

Abbreviations: SD: Standard deviation; BMI: Body mass index; EGD: Esophagogastroduodenoscopy.
